# Chronic Rhinosinusitis—An Update on Epidemiology, Pathogenesis and Management

**DOI:** 10.3390/jcm9072285

**Published:** 2020-07-18

**Authors:** Silviu Albu

**Affiliations:** Iuliu Hatieganu University of Medicine and Pharmacy Cluj-Napoca, 400015 Cluj-Napoca, Romania; silviualbu63@gmail.com

Chronic rhinosinusitis (CRS) is one of the most common chronic medical conditions worldwide, affecting all age groups. Its estimated incidence is 12.3% in the USA, 10.9% in Europe and 13% in China [1,2,3]. CRS is also a condition leading to a significant decrease in the quality of life of patients [4]. It has been demonstrated that CRS had a greater effect on social function that ischemic heart disease or chronic heart failure [5].

Furthermore, it is documented that CRS is also an economic burden for society. It is estimated that in the USA, the direct costs associated with CRS are approximately USD 10–13 billion per year [6]. Moreover, indirect costs of CRS are due to missed workdays, absenteeism and productivity loss, and are estimated to exceed USD 20 billion per year in the USA [7].

It is well acknowledged that the Journal of Clinical Medicine (JCM) publishes well-performed basic research and well-conducted clinical studies. As a result of a demanding peer-review process and careful selection process, relevant articles to an extensive readership are issued regularly in the journal. The special issue of JCM, entitled Prevention, Diagnosis and Management of Chronic Rhinosinusitis exemplifies the attempt to deliver high-quality topics relevant for both clinicians and researchers.

*Staphylococcus aureus* is one of the bacteria commonly found in CRS patients, and *S. aureus* biofilms are frequently associated with recalcitrant or recurrent disease [8]. It has been demonstrated that *S. aureus* is able to penetrate the barrier of the columnar epithelium of the paranasal sinuses, thus enhancing the inflammatory process [9,10]. In this issue of JCM, Hu et al. [11] studied the effects of sub-inhibitory clindamycin and azithromycin on the production of *S. aureus* exoproteins and the ensuing effect of decreasing inflammation, epithelial barrier disruption and invasion. The study was performed on primary human nasal epithelial cells (HNECs) from patients undertaking endoscopic skull base techniques without clinical or radiological evidence of CRS. This study emphasizes that *S. aureus* exoproteins induce severe cytotoxicity and interrupt the mucosal barrier, upsetting its function and decreasing inflammation.

The authors demonstrated that sub-inhibitory clindamycin successfully blocked *S. aureus* exoprotein production, subsequently preventing toxicity, reversing the damaging effects on the mucosal barrier architecture and function and modulating its pro-inflammatory features. To a lesser degree, sub-inhibitory azithromycin had comparable effects on these actions. Moreover, the authors demonstrated that subinhibitory clindamycin or azithromycin was able to considerably decrease the *S. aureus* biofilm protein assembly. *S. aureus *treated with clindamycin—but not azithromycin—no longer display an invasive ability on HNECs. This study has important clinical consequences, as these antibiotics might decrease inflammation associated with *S. aureus* biofilm exoproteins.

Understanding the CRS pathophysiology may offer better diagnostic techniques and permit the development of new treatment strategies. The contribution of nitric oxide (NO) in CRS is discussed in the current literature [12,13,14]. Vlad et al. [15] assessed the arginase (ARG) expression in the mucosa of CRS patients. Increased ARG concentration can decrease NO levels by lessening the accessibility of its precursor, L-arginine.

CRS patients expressed significantly higher ARG2 levels as compared to controls. ARG2 levels were significantly increased in CRS without polyps, while in polyposis, ARG2 levels did not reach statistical significance despite showing an increase. CRS without allergy displayed significantly increased levels of ARG2. Compared to controls, ARG2 levels were also significantly increased in non-asthmatic CRS patients and in non-allergic, non-asthmatic CRS patients. In the univariate analysis of CRS patients using the comparisons of allergic vs. non-allergic, asthmatic vs. non-asthmatic, non-asthmatic allergic vs. non-asthmatic non-allergic, and non-allergic asthmatic vs. non-allergic-non-asthmatic, ARG2 levels were statistically higher in non-allergic CRS patients and non-allergic and non-asthmatic patients. The authors suggest that an improved knowledge of ARG functions in CRS will allow the development of personalized treatments.

Kim et al. [16] assessed in their study trends in micro-organisms isolated form CRS patients from Korea, undergoing endoscopic sinus surgery (ESS) in the following periods: 2007–2008, 2011–2012, and 2017–2018. During this time period, the authors undertook endoscopically guided bacterial cultures from the sinus. A total of 510 adult patients were retrospectively reviewed. The bacteria recovery rate was 73.9% for polyposis and 82.8% for CRS without polyps. The analysis demonstrated a significant increase over time in the presence of coagulase negative *Staphylococci* (CNS), *Klebsiella* and *Pseudomonas aeruginosa*. The authors emphasized the significant increasing trend of Gram-negative bacteria isolation in CRS patients. Moreover, extended-spectrum beta-lactamase (ESBL) *Klebsiella* and *Enterobacter* displayed an increasing trend over time. The authors underline the importance of current recommendations of endoscopically guided cultures during ESS. Thus, pathogenic bacteria should be isolated and targeted antibiotics should be prescribed.

Fungal rhinosinusitis is a unique type of CRS with special clinical and pathologic features. In their paper, Lu et al. [17] explore the contribution of bacterial microbiota in different types of CRS. It is well known that conventional culture techniques provide limited evidence regarding bacterial pathogens. On the contrary, next-generation sequencing (NGS), a culture-independent technique, provides a superior illustration of microbiota. The authors undertook 16S rRNA amplification sequencing to discover differences between fungal and non-fungal CRS and between the middle meatus and the nasopharynx. The authors enrolled 7 consecutive fungal and 18 consecutive non-fungal CRS patients in the study. Extracted DNA was analyzed through 16S rRNA amplification. The authors demonstrate that bacterial community diversity was significantly lower in the middle meatus of fungal CRS. Nevertheless, no significant differences were observed between the diversity samples collected from the nasopharynx. *Corynebacterium* and *Fusobacterium* were detected in only non-fungal CRS patients. On the contrary, *Haemophilus* and *Pseudomonas* were both highly prevalent and abundant in fungal CRS. This feature could be associated with bacterial–fungal interaction. The study emphasized that bacterial dysbiosis is more common in fungal CRS and limited to the middle meatus.

This Special Issue of JCM includes two papers on disease-specific form of CRS. The study of Chang et al. [18] examined the occurrence of CRS among patients with Sjögren’s syndrome (SS). A huge database was analyzed: 18,723 SS patients diagnosed between 1997 and 2011 were retrospectively reviewed. A control group of 59,568 patients without SS were matched at the 1:4 ratio to SS patients. Patients were matched by gender, age, income, urban and comorbidities. Comorbidities retrieved included: rhinitis, septal deviation, gastroesophageal reflux disease (GERD) chronic obstructive pulmonary disease (COPD), asthma, diabetes mellitus (DM), and hypertension (HT). The main outcome was CRS occurrence. The authors demonstrated that the cumulative incidence of CRS was statistically increased in the SS group as compared to controls. The Cox proportional model demonstrated that patients with SS have a significant increased CRS incidence (adjusted HR, 2.51; 95% CI, 2.22–2.84; *p* < 0.001). Subgroup analyses of GERD, COPD, asthma, DM, HT, and RA showed that SS is an independent risk factor in the occurrence of CRS. The percentage of patients undergoing surgery in the SS group was significantly lower. Further research is undeniably needed to highlight the features of CRS among SS patients.

The naso-sinusal disease of 64 adult patients with primary ciliary dyskinesia (PCD) is deeply analyzed in the study of Bequignon et al. [19] Among patients included, hearing loss and rhinorrhea were most commonly encountered. Symptom burden was increased in older patients. Abnormal nasal endoscopy was recorded in every case: polyps were present in 33% of patients and sticky, thick, immobile mucus in 87.5% of cases. On the CT scan, sinuses displayed partial opacities, and hypoplasia/agenesis was present in one third of patients. Culture-guided cultures grew *Haemophilus influenzae*, *Streptococcus pneumoniae* and *Pseudomonas aeruginosa*. Otitis media with effusion (OME) was frequently encountered during childhood, but less common in adults. Half of the patients presented sensorineural hearing loss. Ciliary function and ultrastructure were not associated with disease seriousness in the otolaryngology field. The OME occurrence was statistically associated with a forced expiratory volume (FEV1) < 70%. Thus, the existence of OME in adults with PCD could be a marker of severe lung disease.

In the same issue of JCM, Bequignon et al. [20] published another interesting study on the management of CRS in adult patients with PCD. A retrospective review of 41 adult patients with PCD is presented. On the CT scan, partial opacities were described, mainly positioned in the ethmoids and maxillary sinuses. Positive bacteriological cultures were obtained in 83.9% of patients with purulent mucus. Positive cultures for *Pseudomonas aeruginosa* were not significantly different between patients bellow and above 40 years. Surgery was employed in only 19% of patients to improve sinus drainage. The authors emphasize that in PCD patients, sinuses should be considered a bacterial pool. The paper suggests that bacteriology and sinus symptoms rather than CT scans should guide medical and surgical management in this group of patients.

Practical approaches to common problems are also discussed in this issue of JCM. Inverted papilloma (IP) of the maxillary sinus is still considered a challenge for the endoscopic surgeon. In a beautifully illustrated paper, Hildebrand et al. [21] retrospectively review 17 patients with primary or recurrent IP of the maxillary sinus. The prelacrimal endoscopic approach has been used in all patients. No recurrences were described after a median follow-up of 3.8 years. The surgical technique is extensively described and relevant literature on the outcomes of IP surgery is presented to the reader.

Wound healing following ESS remains a significant element for the particular patient. Prolonged inflammation, scarring and synechiae can adversely influence the postoperative outcome. This is the reason why researchers are strongly interested in the generation of materials that may improve postoperative wound healing. Manciula et al. [22] examined in an experimental study the effects of astaxanthin—a powerful antioxidant—on nasal mucosa healing following surgery. The temporal evolution of wound healing was assessed through several parameters evaluating epithelial thickness, subepithelial thickness, goblet cells, and subepithelial fibrosis. The authors clearly demonstrate that astaxanthin given in the postoperative period significantly decreases fibrosis and prevents synechia development.

Wound healing is clinically assessed in a clinical study by Trombitas et al. [23] Middle meatus antrostomy (MMA) stenosis is still reported as an unfavorable outcome in patients undergoing surgery. The authors present a prospective within-subject, randomized, controlled trial assessing the effect of spray cryotherapy on wound healing. Included were 26 patients with bilateral CRS without polyps. The outcomes were represented by MMA diameter and area, histology, and symptoms. The MMA diameter and area were significantly increased in the cryotherapy group. Nasal obstruction and discharge were significantly improved following cryotherapy. Moreover, cryotherapy significantly decreases inflammation, edema and goblet cell hyperplasia.

As demonstrated by the diversity of papers selected in this Special Issue of JCM, there is something interesting for all readers of the journal. Clinicians interested in rhinology, endoscopic surgeons, basic science researchers, public health providers, and internal medicine physicians will find something of interest in this issue. The cautious selection of such articles speaks for the ongoing success of JCM as a leading reference among open access publications.

## References

[1] Hastan D., Fokkens W.J., Bachert C., Newson R.B., Bislimovska J., Bockelbrink A., Bousquet P.J., Brożek G.M., Bruno A., Dahlen S.E. (2011). Chronic rhinosinusitis in Europe—An underestimated disease. A GA(2)LEN study. Allergy.

[2] Palmer J.N., Messina J.C., Biletch R., Grosel K., Mahmoud R.A. (2019). A cross-sectional, population- based survey of U.S. adults with symptoms of chronic rhinosinusitis. Allergy Asthma Proc..

[3] Hirsch A.G., Stewart W.F., Sundaresan A.S., Young A.J., Kennedy T.L., Greene J.S., Feng W., Tan B.K., Schleimer R.P., Kern R.C. (2017). Nasal and sinus symptoms and chronic rhinosinusitis in a population-based sample. Allergy.

[4] Schlosser R.J., Gage S.E., Kohli P., Soler Z.M. (2016). Burden of illness: A systematic review of depression in chronic rhinosinusitis. Am. J. Rhinol. Allergy.

[5] Gliklich R.E., Metson R. (1995). The health impact of chronic sinusitis in patients seeking otolaryngologic care. Otolaryngol. Head Neck Surg..

[6] Rudmik L. (2017). Economics of chronic rhinosinusitis. Curr. Allergy Asthma Rep..

[7] Bhattacharyya N. (2011). Incremental health care utilization and expenditures for chronic rhinosinusitis in the United States. Ann. Otol. Rhinol. Laryngol..

[8] Foreman A., Jervis-Bardy J., Wormald P.J. (2011). Do biofilms contribute to the initiation and recalcitrance of chronic rhinosinusitis?. Laryngoscope.

[9] Ou J., Drilling A., Sinqhal D., Tan N.C., Wallis-Hill D., Vreuqde S., Psaltis A.J., Wormald P.J. (2016). Association of intracellular *Staphylococcus aureus* with prognosis in chronic rhinosinusitis. Int. Forum Allergy Rhinol..

[10] Tan N.C., Cooksiey C.M., Roscioli E., Douqlas R., Wormald P.J., Vreuqde S. (2014). Small-colony variants and phenotype switching of intracellular *Staphylococcus aureus* in chronic rhinosinusitis. Allergy.

[11] Hu H., Ramezanpour M., Hayes A.J., Liu S., Psaltis A.J., Wormald P.J., Vreugde S.J. (2019). Sub-inhibitory clindamycin and azithromycin reduce S. aureus exoprotein induced toxicity, inflammation, barrier disruption and invasion. Clin. Med..

[12] Eby G.A. (2006). Strong humming for one hour daily to terminate chronic rhinosinusitis in four days: A case report and hypothesis for action by stimulation of endogenous nasal nitric oxide production. Med. Hypotheses.

[13] Taruya T., Takeno S., Kubota K., Sasaki A., Ishino T., Hirakawa K. (2015). Comparison of arginase isoform expression in patients with different subtypes of chronic rhinosinusitis. J. Laryngol. Otol..

[14] Bommarito L., Guida G., Heffler E., Badiu I., Nebiolo F., Usai A., De Stefani A., Rolla G. (2008). Nasal nitric oxide concentration in suspected chronic rhinosinusitis. Ann. Allergy Asthma Immunol..

[15] Vlad D., Albu S. (2019). Arginase isoform expression in chronic rhinosinusitis. J. Clin. Med..

[16] Kim D., Assiri A.M., Kim J.H. (2019). Recent trends in bacteriology of adult patients with chronic rhinosinusitis. J. Clin. Med..

[17] Lu Y.T., Wang S.H., Liou M.L., Shen T.A., Lu Y.C., Hsin C.H., Yang S.F., Chen Y.Y., Chang T.H. (2019). Microbiota dysbiosis in fungal rhinosinusitis. J. Clin. Med..

[18] Chang G.H., Chen Y.C., Lin K.M., Yang Y.H., Liu C.Y., Lin M.H., Wu C.Y., Hsu C.M., Tsai M.S. (2019). Real-world database examining the association between Sjögren’s syndrome and chronic rhinosinusitis. J. Clin. Med..

[19] Bequignon E., Dupuy L., Zerah-Lancner F., Bassinet L., Honoré I., Legendre M., Devars du Mayne M., Escabasse V., Crestani B., Maître B. (2019). Critical evaluation of sinonasal disease in 64 adults with primary ciliary dyskinesia. J. Clin. Med..

[20] Bequignon E., Dupuy L., Escabasse V., Zerah-Lancner F., Bassinet L., Honoré I., Legendre M., Devars du Mayne M., Crestani B., Escudier E. (2019). Follow-up and management of chronic rhinosinusitis in adults with primary ciliary dyskinesia: Review and experience of our reference centers. J. Clin. Med..

[21] Hildenbrand T., Weber R., Mertens J., Stuck B.A., Hoch S., Giotakis E. (2019). Surgery of inverted papilloma of the maxillary sinus via translacrimal approach-long-term outcome and literature review. J. Clin. Med..

[22] Manciula L.G., Berce C., Tabaran F., Trombitaș V., Albu S. (2019). The effects of postoperative astaxanthin administration on nasal mucosa wound healing. J. Clin. Med..

[23] Trombitaș V., Zolog A., Toader M., Albu S. (2019). Maxillary antrostomy patency following intraoperative use of spray cryotherapy. J. Clin. Med..

